# Effect of hydroxypropyl starch on the multiscale structure and pasting properties of starch in *γ*-irradiated fresh rice noodles

**DOI:** 10.3389/fnut.2026.1825187

**Published:** 2026-06-26

**Authors:** Ruyue Deng, Zhiqiang Yan, Danqiu Luo, Huihui Tang, Jianqiang Wu, Chaochao Zheng, Shengwei Wang

**Affiliations:** 1 Guizhou Rice Research Institute, Guizhou Academy of Agricultural Sciences, Guiyang, China; 2 College of Chinese Medicine and Health, Guizhou University of Traditional Chinese Medicine, Guiyang, China

**Keywords:** amylopectin structure, fresh rice noodles, gamma irradiation, hydroxypropyl starch, pasting properties

## Abstract

Fresh rice noodles (FRNs) are widely consumed starch based foods whose quality and shelf life depend strongly on the structural stability of the starch matrix. *γ*-Irradiation can effectively extend the shelf life of FRNs but induces starch depolymerization, altering starch structure and functionality. This study investigated the effects of hydroxypropyl starch (HPS) incorporation on the molecular characteristics, ordered structure, and thermal and pasting properties of starch in *γ*-irradiated FRNs. The results showed that 6% HPS incorporation produced the most balanced structural and functional characteristics. Overall, HPS increased molecular size parameters, apparent *α*-1,6 linkage ratio, short range molecular order, relative crystallinity, and peak viscosity, while decreasing the long B3 chains proportion and gelatinization enthalpy. Correlation analysis showed that A chains were positively correlated with short-range order, relative crystallinity, and swelling power, and negatively correlated with peak viscosity, whereas B3 chains showed the opposite trend, suggesting that the superior pasting behavior at 6% HPS was associated with a more balanced amylopectin chain architecture. HPS incorporation altered the relative amylopectin chain length distribution of the starch system, with 6% addition increasing the proportion of A and B1 chains relative to B2 and B3 chains, thereby favoring granule swelling and paste viscosity development. However, excessive HPS addition over-enriched short chains and weakened paste stability. These findings clarify the structure–function relationships in *γ*-irradiated starch systems and provide a scientific basis for optimizing the quality of irradiated rice-based products.

## Graphical abstract

**Figure:**
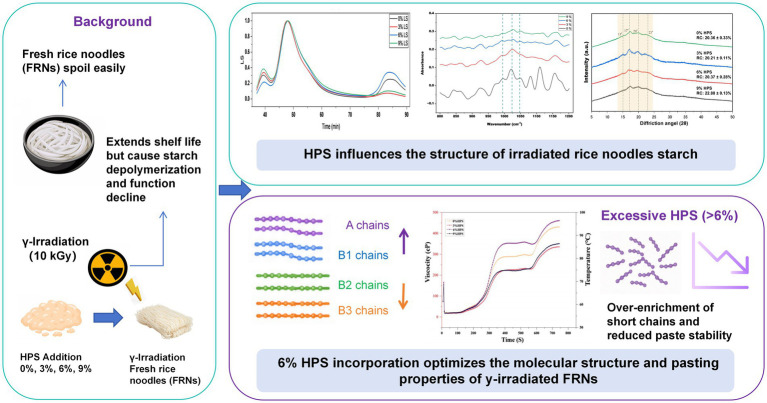

## 1 Introduction

Fresh rice noodles (FRNs) are globally favored owing to desirable chewiness and smooth texture (1). However, the shelf life of FRNs is severely limited by their high susceptibility to microbial spoilage, which is primarily attributed to a high moisture content (2–4). Gamma (*γ*)-irradiation is widely used as an effective non-thermal sterilization technology (5). Despite its efficacy, the widespread application of *γ*-irradiation in FRNs is limited by the unavoidable radiolytic degradation of starch (6). Specifically, *γ*-irradiation modifies starch structure primarily through free radical generation and chain scission. These reactive species preferentially cleave glycosidic bonds in amorphous regions, leading to molecular depolymerization, reduced molecular weight, and increased structural disorder (7, 8). Meanwhile, partial rearrangement within crystalline regions may occur, generally accompanied by a decrease in overall crystallinity. These structural changes typically result in reduced thermal stability, altered gelatinization enthalpy, and viscosity, thereby affecting processing properties (9).

Hydroxypropyl starch (HPS) is a chemically modified starch characterized by hydroxypropyl substitution, which affects intermolecular hydrogen bonding and enhances molecular flexibility and water-binding capacity (10, 11). Owing to these properties, HPS has been widely employed to improve the stability of starch-based foods (11–13). Previous studies have demonstrated that hydroxypropylated starch can maintain quality during frozen storage by inhibiting chain reassociation and modifying hydration behavior (14). Compared with non-starch hydrocolloids, HPS as a starch derivative, exhibits better compositional compatibility with the noodle matrix and can more directly regulate starch–starch and starch–water interactions, thereby helping to mitigate irradiation-induced damage to starch structure. From an industrial perspective, the combination of *γ*-irradiation with modified starch may provide a practical basis for improving the quality stability of fresh rice noodles during non-thermal preservation. These functional advantages suggest that HPS may serve as a potential structural regulator in starch systems subjected to physical stress.

Accordingly, HPS may also affect starch organization in a *γ*-irradiated FRN matrix, where the bulky hydroxypropyl groups may introduce steric hindrance (15). Consequently, the disordered aggregation of radiation-cleaved short chains could be suppressed. Furthermore, the strong water-binding capacity of HPS may alter the moisture distribution within the matrix, thereby buffering the radiation-induced damage to the semicrystalline lamellae of native starch (3, 4, 16). These steric and hydration effects suggest that HPS may influence the multi-scale structure of starch and, consequently, its pasting behavior under irradiation.

Although previous studies have established the effects of *γ*-irradiation on starch structure and functionality, most have focused on irradiation dose or differences among starches from various cereal sources (17, 18). By comparison, research on HPS has mainly concerned its preparation, application in biobased packaging films, and effects on the cooking properties and shelf life of starch-based foods (13, 19). However, whether HPS can modulate irradiation-induced multi-scale structural changes in starch and the associated pasting behavior in fresh rice noodles has not been systematically investigated.

Therefore, the present study aimed to investigate the effects of hydroxypropyl starch on the multi-scale structural characteristics and pasting properties of starch isolated from *γ*-irradiated fresh rice noodles. The findings are expected to deepen understanding of the regulatory role of HPS in starch systems under radiation stress and provide theoretical guidance for the development of high-quality irradiated FRN products.

## 2 Materials and methods

### 2.1 Materials and reagents

Milled rice (cv. *Iyou 4,716*) used in this study was harvested in 2024 and supplied by Guizhou Rice Research Institute (Guiyang, China). Food-grade hydroxypropyl starch (HPS, degree of substitution, DS = 0.12) was purchased from Henan Hengrui Starch Technology Co., Ltd., (Luohe, China). Dimethyl sulfoxide (DMSO, ≥99.5%) and oligosaccharides kit were purchased from Sigma-Aldrich (Sigma, St. Louis, MA, USA). Isoamylase (EC 3.2.1.68) and protease (EC 3.4.21.62) were supplied by Megazyme Co., Ltd. (Wicklow, Ireland). All other chemicals and reagents were of analytical grade and purchased from Aladdin Biochemical Technology Co., Ltd. (Shanghai, China).

### 2.2 Preparation of *γ*-irradiated fresh rice noodles with hydroxypropyl starch

Polished rice was soaked (12 h, rice-to-water ratio of 1: 1), wet-milled, centrifuged (3,000 g, 10 min), and the sediment was air-dried at 40 °C, ground, and sieved through an 80-mesh screen to obtain rice flour. Rice flour was mixed with water at a 1: 1 mass ratio to form a batter, with HPS added at 0, 3, 6, and 9% (w/w, based on rice flour). After standing at room temperature for 20 min, the batter was homogenized, evenly spread onto flat containers, and steamed at 100 °C for 8 min to obtain fresh rice noodles. The noodles were cooled to 25 °C, vacuum-packed in polyethylene pouches (250 g per bag), and subjected to *γ*-irradiation at 10 kGy using a ^60^Co source at room temperature, with a dose rate of 2 kGy/h for 5 h.

### 2.3 Isolation of starch from *γ*-irradiated fresh rice noodles

Samples were dispersed in double-distilled water (ddH₂O), mixed with DMSO (1: 10, w/v), and heated in a boiling water bath for 2 h. After centrifugation (5,000 g, 10 min), starch was precipitated from the supernatant with eight volumes of absolute ethanol (2 h, room temperature) and collected by centrifugation (5,000 g, 15 min). The precipitates were washed three times with ddH₂O, dried at 40 °C to constant weight, ground, passed through a 100-mesh sieve, and stored at 4 °C until analysis.

### 2.4 Molecular weight distribution by SEC–MALLS

Molecular weight distribution was determined using size-exclusion chromatography coupled with multi-angle laser light scattering and refractive index detection (SEC-MALLS-RI). Starch (5 mg, dry basis) was dissolved in 5 mL DMSO/LiBr (0.5%, w/w) at 80 °C for 3 h prior to injection. The system was equipped with a DAWN HELEOS II multi-angle laser photometer (He-Ne laser, *λ* = 663.7 nm, Wyatt Technology, Santa Barbara, CA, USA), a refractive index detector, and three Shodex OHpak SB columns in series (SB-805, SB-804, SB-803, 300 × 8 mm, Showa Denko K. K., Tokyo, Japan). The columns were maintained at 60 °C with DMSO/LiBr (0.5%, w/w) as mobile phase at a flow rate of 0.3 mL/min. The dn/dc value was taken as 0.07 mL/g. Weight-average molecular weight (Mw), number-average molecular weight (Mn), and polydispersity index (Mw/Mn) were calculated using ASTRA 6.1 software (Wyatt Technology).

### 2.5 Chain-length distribution by HPAEC-PAD

The analysis was performed using the reported method with minor modifications (20). Starch (10 mg) was gelatinized in boiling water for 60 min, debranched with isoamylase (1,400 U) at 37 °C for 24 h, and reduced with sodium borohydride for 20 h. Samples were dried under nitrogen, dissolved in 1 M NaOH, diluted, and centrifuged (12,000 g, 5 min) before injection. Analysis was performed on an ICS 5000 + system equipped with a CarboPac PA200 column (250 × 4.0 mm) and pulsed amperometric detector. The mobile phase consisted of eluent A (0.2 M NaOH) and eluent B (0.2 M NaOH/0.2 M sodium acetate). The column temperature was set at 30 °C, the flow rate was 0.4 mL/min, and the injection volume was 5 μL. The elution gradient was programmed as follows: 90: 10 (A: B) from 0 to 10 min, a linear gradient to 40: 60 from 10 to 30 min, and 40: 60 from 30 to 50 min. Maltooligosaccharide standards were used for DP assignment, and the relative area percentage of DP 6–76 was calculated.

### 2.6 Degree of branching by ^1^H NMR

Degree of branching (DB) was determined by ^1^H NMR. Starch (5 mg, dry basis) was dissolved in 1 mL DMSO-d₆ at 80 °C overnight and centrifuged (11,000 g, 10 min) before analysis. ^1^H NMR spectra were recorded on a 500 MHz spectrometer (Bruker BioSpin GmbH, Germany) with 32 scans. Peak areas of *α*-1,6 and α-1,4 linkages were integrated using MestReNova software, and DB (%) was calculated as A/(A + B) × 100%.

### 2.7 Fourier transform infrared (FTIR) spectroscopy

Fourier transform infrared (FTIR) spectra of starch were recorded using a Nicolet iZ10 spectrometer (Thermo Fisher Scientific, Waltham, MA, USA). Dried starch sieved through a 100-mesh sieve was mixed with dry KBr and pressed into transparent pellets. Spectra were collected over 4,000–400 cm^−1^ with a resolution of 4 cm^−1^ and 32 scans. The absorbance ratio at 1047/1022 cm^−1^ was used as an indicator of short-range order in the starch structure.

### 2.8 Crystalline structure by X-ray diffraction (XRD)

Dried starch passed through a 100-mesh sieve was formed into a flat, compact specimen. XRD patterns were collected using a diffractometer (MiniFlex 600, Rigaku, Tokyo, Japan) with Cu Kα radiation (*λ* = 1.5406 Å) operated at 40 kV and 600 W. Diffraction intensity was recorded from 4° to 60° (2θ) with a step size of 0.01° and a scanning speed of 5°/min. Diffractograms were analyzed with MDI Jade 5.0 to determine relative crystallinity, crystalline type and characteristic diffraction peak positions (2θ).

### 2.9 Morphological observation by scanning electron microscopy (SEM)

Dried starch was gently ground and passed through a 100-mesh sieve, then washed three times with 4% (w/v) SDS solution and five times with ddH₂O, and finally resuspended in absolute ethanol. A small aliquot of the ethanol suspension was deposited onto metal stubs covered with double-sided conductive carbon tape and dried at 37 °C overnight. The samples were sputter-coated with gold and observed using a field-emission SEM (Merlin Compact, Carl Zeiss, Germany) operated at 3 kV. Micrographs were recorded at magnifications of 1,000–5,000 × .

### 2.10 Differential scanning calorimetry (DSC)

Gelatinization properties of starch samples were determined by differential scanning calorimetry (DSC). Ground samples sieved through a 100-mesh screen were weighed into aluminum pans, deionized water was added, and the pans were sealed and equilibrated at 4 °C for 24 h. Measurements were performed using a DSC 200 F3 (NETZSCH, Selb, Germany) by heating from 30 to 105 °C at 10 °C/min. An empty sealed aluminum pan was used as reference. Thermograms were analyzed with Proteus Thermal Analysis software to obtain the onset temperature (To), peak temperature (Tp), conclusion temperature (Tc) and gelatinization enthalpy (ΔH).

### 2.11 Pasting properties by rapid visco analyzer (RVA)

Pasting properties of the samples were determined using a rapid visco analyzer (RVA Super 4, Newport Scientific, Warriewood, Australia). Samples equilibrated to constant moisture were ground and passed through a 100-mesh sieve. Moisture content was determined, and the required sample mass for RVA analysis (on a constant-moisture basis) was weighed into an aluminum canister. Deionized water (25 g) was added, and the suspension was mixed thoroughly.

The suspension was stirred at 960 rpm for the first 10 s and then at 160 rpm for the remainder of the test. The temperature profile consisted of holding at 50 °C for 1 min, heating to 95 °C, holding, and then cooling to 50 °C and holding for 2 min, giving a total test time of 13 min. Viscosity curves were recorded and analysed using TCW3 software to obtain peak viscosity, trough viscosity, breakdown, final viscosity, setback, peak time and pasting temperature. Breakdown was calculated as peak viscosity minus trough viscosity, and setback as final viscosity minus trough viscosity.

### 2.12 Statistical analysis

All experiments were performed at least in triplicate, and the results are expressed as means ± standard deviation. Statistical analyses were conducted using SPSS 26.0 (IBM Corp., Armonk, NY, USA). Differences among group means were evaluated by one-way ANOVA followed by Duncan’s multiple range test. Values in the same column with different superscript letters were considered significantly different at *p* < 0.05.

## 3 Results and discussion

### 3.1 Basic physicochemical properties

The basic physicochemical properties of rice starch with different levels of hydroxypropyl starch (HPS) are shown in Table 1. Total starch content increased significantly from 73.18 to 82.77% with increasing HPS addition (*p* < 0.05), whereas apparent amylose content (AAC) decreased from 18.53 to 15.44%. Both water solubility index (WSI) and swelling power (SP) exhibited significant concentration-dependent increases, with WSI increasing from 22.38 to 24.76% and SP from 11.50 to 12.97 g/g (*p* < 0.05).

**Table 1:** Basic physicochemical properties of rice starch with different hydroxypropyl starch (HPS) incorporation levels.

Samples	Total starch content (%)	Apparent amylose content (%)	WSI (%)	SP (g/g)
0% HPS	73.18 ± 0.27^d^	18.53 ± 0.18^a^	22.38 ± 0.34^c^	11.50 ± 0.21^b^
3% HPS	75.63 ± 0.30^c^	17.58 ± 0.24^b^	23.07 ± 0.13^b^	11.95 ± 0.18^b^
6% HPS	79.99 ± 0.21^b^	16.67 ± 0.41^c^	24.22 ± 0.33^a^	12.36 ± 0.35^a^
9% HPS	82.77 ± 0.40^a^	15.44 ± 0.17^d^	24.76 ± 0.10^a^	12.97 ± 0.29^a^

WSI, Water solubility index; SP, Swelling power. Values are mean ± SD (*n* = 3). Different superscript letters within the same column indicate significant differences (*p* < 0.05).

The decline in AAC reflects altered iodine-binding capacity. Irradiation-induced chain scission may shorten linear glucan segments, while hydroxypropyl substitution introduces steric hindrance that interferes with iodine complex formation (21). The increased WSI and SP indicate enhanced hydration of the starch matrix. This is attributed to the hydroxypropyl groups weaken inter-chain hydrogen bonding, facilitating water penetration, granule swelling, and polymer solubilization (22). Collectively, these results indicate that HPS modifies the molecular organization and hydration behavior of irradiated starch, which may subsequently influence its structural ordering and gelatinization behavior.

### 3.2 Molecular structure

#### 3.2.1 Molecular weight distribution

**Figure 1:**
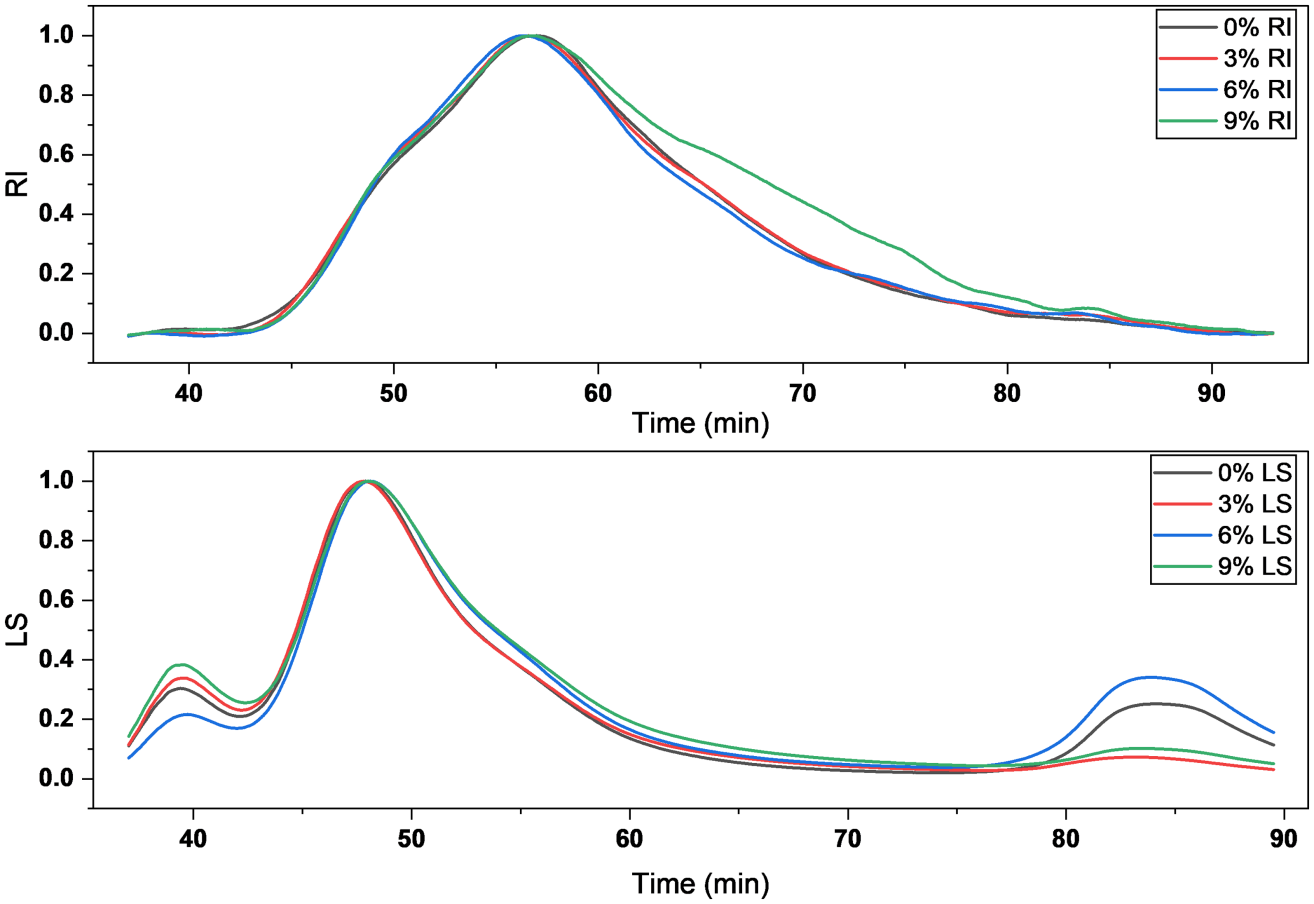
SEC-LLS spectra of starch with different HPS additions.

Figure 1 displays the molecular characterization profiles of starch in irradiated rice noodles with varying proportions of hydroxypropyl starch. The RI results showed no significant shift in the main peak, indicating that HPS addition did not affect the main molecular components of starch. In contrast, the LS signal along with molecular parameters (Table 2) reveal a redistribution of molecular characteristics. The enhanced early-elution signal and weakened late-elution peak at 9% HPS indicate an increased contribution of large molecular species, consistent with the significant increases in Mw and Rn. Conversely, the 6% sample showed a higher proportion of small molecules, in agreement with its relatively lower Mw and unchanged Rn. HPS incorporation significantly increased Mn, Mw, Mp, and Mz, with the highest values observed at 9% (*p* < 0.05). Compared with the control, Mn and Mw increased by 1.73-fold and 1.41-fold, respectively, while polydispersity decreased, indicating a more uniform molecular distribution. The Rn also increased markedly, suggesting enhanced molecular expansion.

**Table 2:** Molecular parameters of *γ*-irradiated rice starches with different HPS additions.

Sample	Mn/kDa	Mp/kDa	Mw/kDa	Mz/kDa	Mw/Mn	Rn/nm
0% HPS	412.966 ± 40.33^c^	415.689 ± 17.82^d^	807.549 ± 12.14^d^	2178.601 ± 52.23^b^	1.967 ± 0.17^a^	51.795 ± 7.68^b^
3% HPS	558.892 ± 7.23^b^	520.53 ± 5.92^b^	953.947 ± 18.19^b^	2280.794 ± 77.74^b^	1.707 ± 0.03^b^	56.096 ± 8.27^b^
6% HPS	529.935 ± 11.74^b^	473.724 ± 1.87^c^	875.607 ± 10.71^c^	1955.839 ± 25.12^c^	1.653 ± 0.02^b^	54.959 ± 7.75^b^
9% HPS	712.998 ± 22.44^a^	690.864 ± 11.74^a^	1141.326 ± 32.52^a^	2793.649 ± 135.46^a^	1.634 ± 0.06^b^	88.155 ± 9.47^a^

Mn, Number average molecular weight. Mp, Peak molecular weight. Mw, Weight average molecular weight. Mz, Average molecular weight of z. Mw/Mn, Polydispersity index. Rn, Radius of Gyration. Different lower case letters show significant difference in the same column.

These changes arise from the interplay between irradiation-induced depolymerization and HPS-mediated chain reorganization (23, 24). Hydroxypropyl groups enhance chain hydration and hydrogen bonding, promoting reassociation of degraded chains, while steric effects limit close packing (25). At low HPS content (3%), the increased hydration associated with hydroxypropyl groups may facilitate chain reassociation, while the relatively low degree of substitution imposes limited steric hindrance (21). This allows molecular chains to recombine into relatively compact structures, as reflected by an increase in Mw with stable Rn. At 6% HPS, greater hydroxypropyl substitution introduced into the starch system leads to increased steric hindrance, which partially limits intermolecular reassociation (26, 27). Consequently, the overall chain conformation shows no significant expansion, and the LS profile exhibits a smaller apparent molecular size. At 9% HPS, the high degree of substitution enhances chain hydration while introducing substantial steric effects, leading to the formation of loosely associated aggregates from degraded chains. These expanded and less compact structures exhibit a larger apparent molecular size, as reflected by the dominance of high-molecular-weight fractions in the LS profile, accompanied by increases in both Mw and Rn (28). Overall, 6% HPS represents a transition point between reassociation, steric and hydration effects, whereas higher substitution shifts the system toward expansion-dominated structures (21).

#### 3.2.2 Chain length distribution

**Figure 2:**
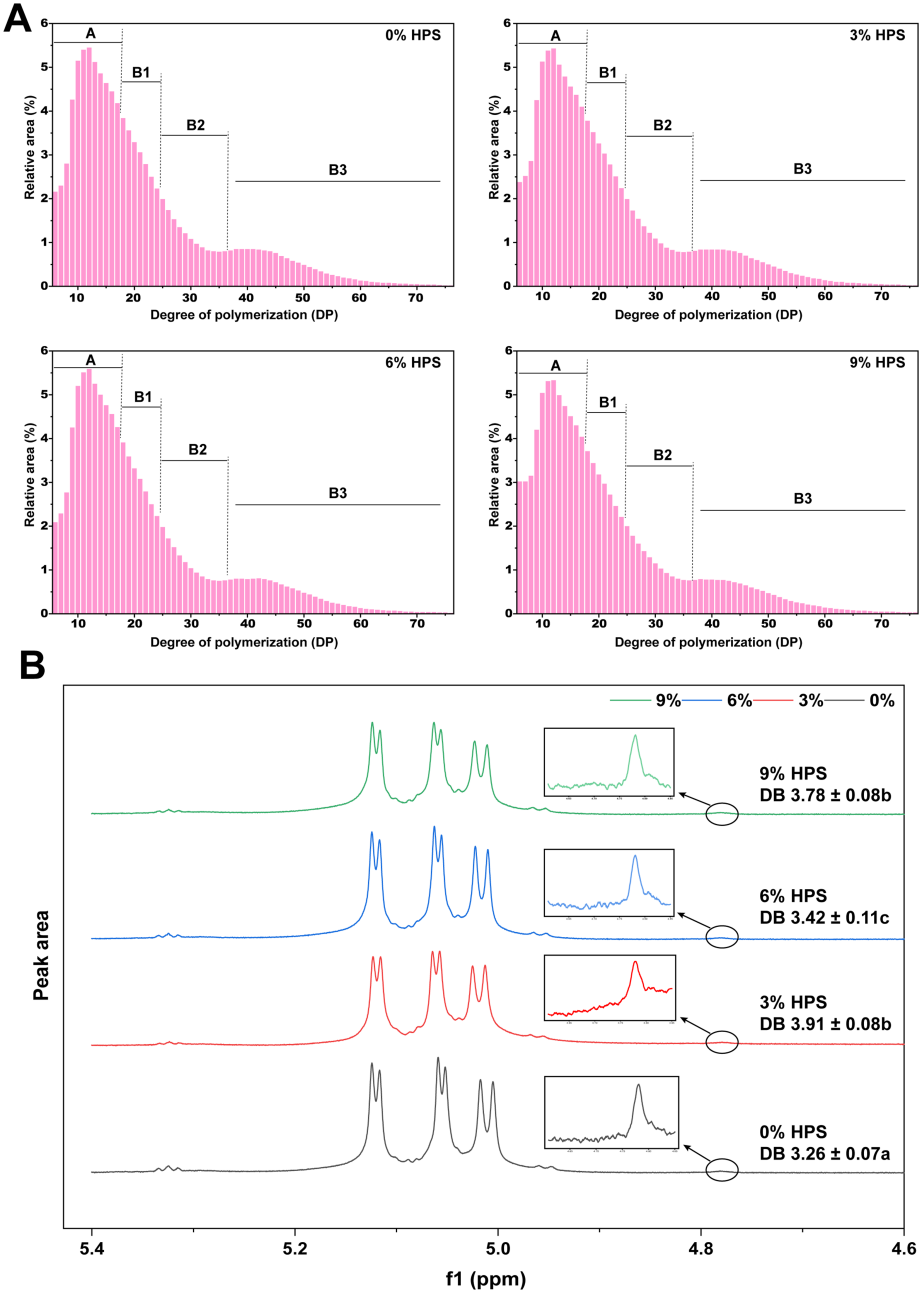
Chain length distribution **(A)** and branching degree **(B)** of starch with different HPS additions.

HPS incorporation significantly altered the branch chain distribution of irradiated starch (Figure 2A and Table 3). At 9% HPS, the proportion of A chains (DP 6–12) increased to 29.07%. In contrast, the proportion of B3 chains (DP ≥ 37) decreased progressively. The distribution of intermediate chains showed distinct variations among treatments. The proportion of B1 chains (DP 13–24) increased and reached the highest value at 6% HPS, whereas B2 chains (DP 25–36) showed a slight decrease at 6% HPS followed by a significant increase at 9% HPS. These results indicate that HPS addition caused a redistribution of amylopectin branch chains in the irradiated starch system.

**Table 3:** Chain length distribution of *γ*-irradiated rice starches with different HPS additions.

Samples	A chains (DP 6–12)	B1 chains (DP 13–24)	B2 chains (DP 25–36)	B3 chains (DP ≥ 37)
0% HPS	27.56% ± 0.02%^d^	44.51% ± 0.05%^b^	13.95% ± 0.04%^c^	13.98% ± 0.02%^b^
3% HPS	27.94% ± 0.02%^b^	44.01% ± 0.02%^c^	14.01% ± 0.01%^b^	14.04% ± 0.03%^a^
6% HPS	27.68% ± 0.03%^c^	45.23% ± 0.01%^a^	13.60% ± 0.02%^d^	13.49% ± 0.03%^c^
9% HPS	29.07% ± 0.01%^a^	43.23% ± 0.03%^d^	14.39% ± 0.01%^a^	13.31% ± 0.02%^d^

Values are mean ± SD. Different superscripts within the same column indicate significant differences (*p* < 0.05).

The observed changes suggest a shift in amylopectin cluster architecture toward shorter branch segments with increasing HPS level. The reduction in B3 chains implies weakened long-range connectivity among adjacent clusters, while the increase in A chains indicates a greater proportion of short branches with higher conformational mobility, consistent with the molecular expansion observed in Section 3.2.1 (29).

Notably, the highest proportion of B1 chains observed at 6% HPS suggests a relatively even redistribution of branch populations. B1 chains mainly contribute to intra-cluster connectivity in amylopectin, and their enrichment may promote granule swelling while maintaining the fundamental organization of the cluster structure (30, 31). In contrast, the marked increase in A chains together with the reduction of B1 and B3 chains at 9% HPS indicates a less integrated cluster architecture (32). Such structural changes may influence the hydration behavior and thermal response of starch during processing (33).

#### 3.2.3 Degree of branching

The ^1^H NMR spectra exhibited characteristic signals of *α*-1,4 and α-1,6 glycosidic linkages at approximately *δ* 5.12 ppm and δ 4.78 ppm, respectively (Figure 2B). No noticeable chemical shift displacement or additional resonances were observed upon HPS incorporation, indicating that the primary glycosidic framework of irradiated starch remained unchanged. However, variations in the relative intensity of the α-1,6 signal were evident. Quantitative analysis showed that the DB values were significantly higher than those of the control (*p* < 0.05), indicating an increased relative proportion of α-1,6 linkages.

Given that *γ*-irradiation preferentially cleaves α-1,4 glycosidic bonds while α-1,6 linkages are comparatively resistant, the increase in DB is more likely attributable to preferential shortening of linear segments, resulting in a relative enrichment of branch linkages (23, 24). This interpretation aligns with the chain-length distribution results, which showed increased short A chains and decreased long B3 chains.

In addition, hydroxypropyl groups exert steric hindrance that disrupts intermolecular hydrogen bonding, thus reducing the tight packing of linear glucan chains. This results in a more spatially dispersed molecular organization and contributes to higher apparent branching complexity. In irradiated starch systems, these steric and hydrogen-bonding effects may further alter the reconstruction behavior of fragmented chains (34). At intermediate substitution levels (6% HPS), steric hindrance from hydroxypropyl groups becomes more pronounced, disrupting the arrangement of glucan chains and leading to a decrease in DB relative to 3% HPS (35, 36). At 9% HPS, enhanced intermolecular interactions promote chain reassociation, while steric hindrance prevents tight packing, resulting in loosely associated aggregates and a more pronounced contribution of *α*-1,6 linkages, leading to an increase in DB. This trend is consistent with the variation in molecular size associated with steric hindrance-regulated chain reassociation. The structural features observed at 6% HPS suggest a more balanced organization, whereas higher HPS levels may result in a relatively over-dispersed structure.

### 3.3 Ordered structure

#### 3.3.1 Short-range ordered structure

**Figure 3:**
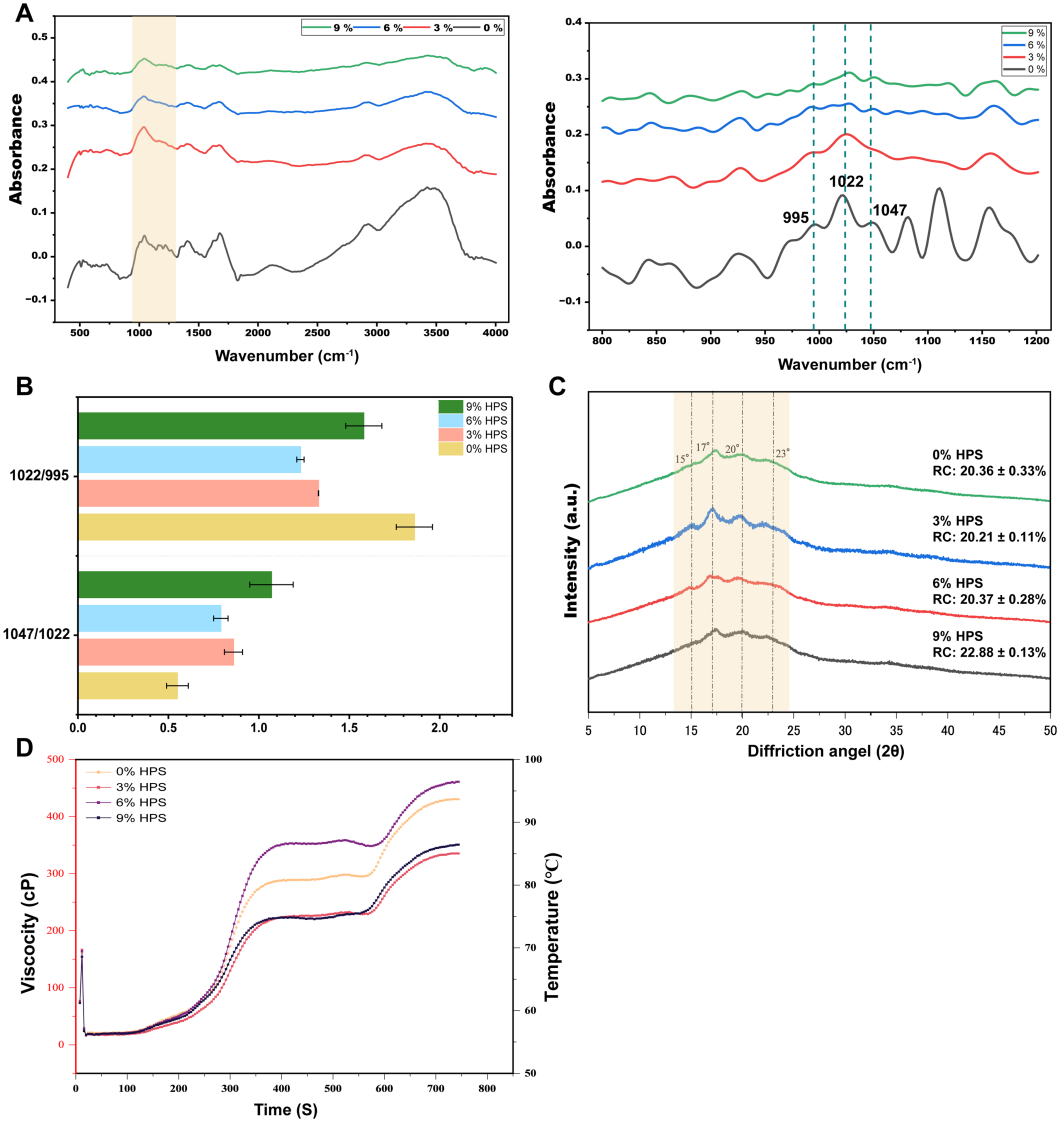
FTIR spectra **(A)**, Fixed wavelength ratio **(B)**, XRD spectra **(C)** and pasting profiles **(D)** of starch with different HPS additions.

The FTIR spectra (Figure 3A) of all samples exhibited typical starch absorption patterns with no new functional groups observed, indicating that HPS incorporation did not alter the primary chemical framework of irradiated starch. Structural differences were mainly reflected in the relative intensities within the short-range ordered region (1200–900 cm^−1^). The absorbance ratio at 1047/1022 cm^−1^ increased significantly with HPS addition, from 0.55 in the control to 1.07 at 9% HPS (*p* < 0.05), indicating enhanced short-range molecular order. Meanwhile, the 1022/995 cm^−1^ ratio decreased overall compared with the control, suggesting a reduction in amorphous structural features.

*γ*-irradiation preferentially cleaves α-1,4 glycosidic linkages, generating shorter amylopectin chains with increased conformational mobility, thereby creating structural conditions for molecular reassociation. At moderate HPS levels, the spatially dispersed but not excessively expanded molecular arrangement allows reassociating chains to achieve favorable intermolecular proximity (37), thereby stabilizing transient double-helical junctions and promoting short-range ordering (29, 38). However, at higher HPS levels, the further expanded intermolecular spacing may promote the formation of locally ordered domains while reducing the cooperativity among adjacent ordered regions, potentially limiting the overall structural stability of the short-range ordered network (39, 40).

#### 3.3.2 Crystalline structure

The X-ray diffraction patterns (Figure 3C) exhibited typical A-type crystalline characteristics, with major diffraction peaks at approximately 15°, 17°, and 23° (2θ). The diffraction features observed at approximately 20° may include contributions from V-type structures. This could be associated with the formation of single-helical inclusion complexes involving amylose and small molecular components. The peaks position remained unchanged, indicating that the crystalline polymorph of rice starch was not altered, while the relative crystallinity increased significantly at 9% HPS. The increased proportion of short amylopectin chains likely facilitates the formation of double helices that assemble into crystalline lamellae. Notably, high HPS content may increase intermolecular spacing through steric hindrance effects. While this may promote ordered stacking, it potentially limits the packing density and stability of the crystalline domains. A comparable phenomenon has been reported for hydroxypropylated-extruded sorghum starch, in which gelatinization temperature and enthalpy decreased simultaneously, suggesting the formation of less thermally stable crystalline domains (41).

### 3.4 Granule morphology

**Figure 4:**
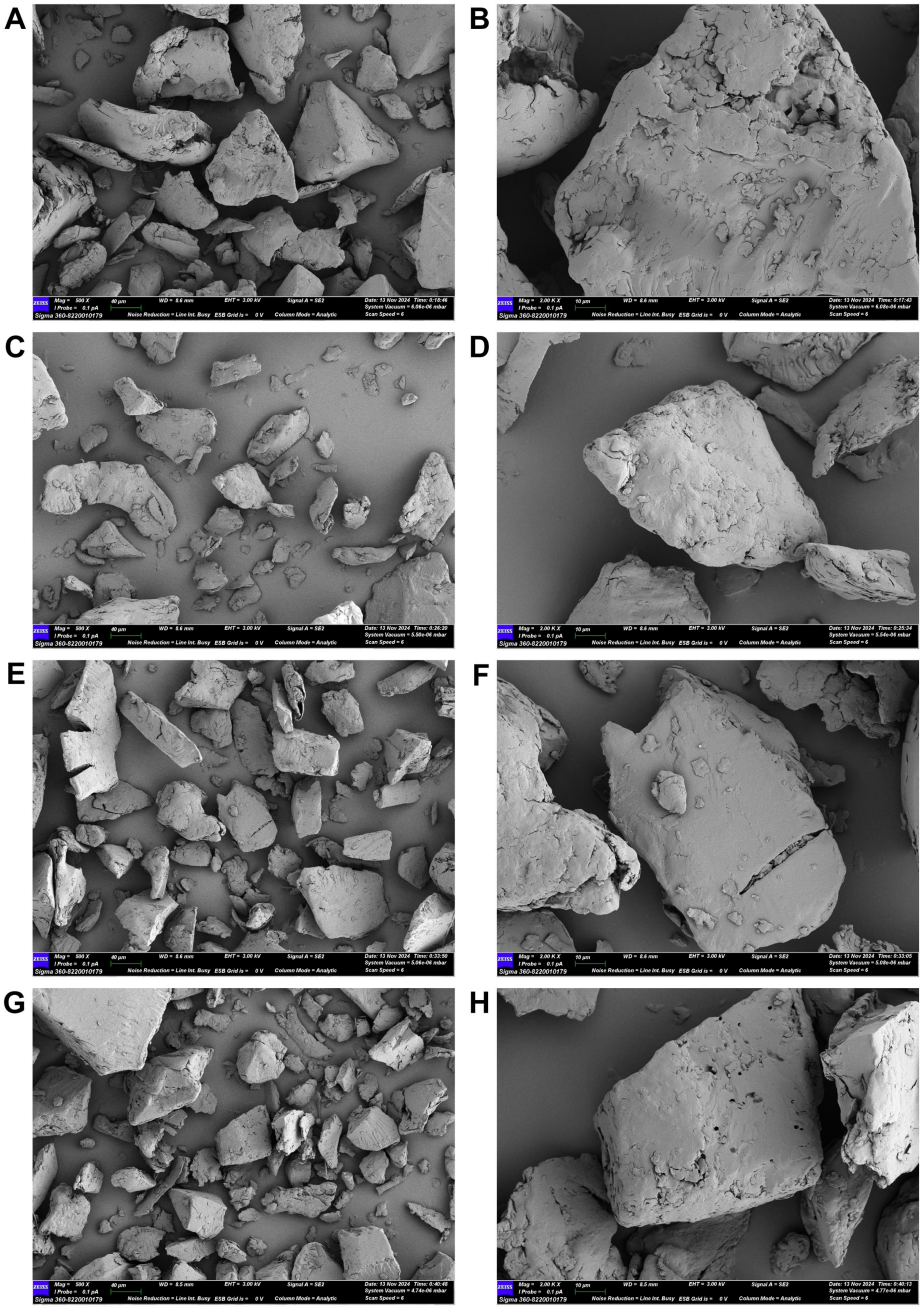
SEM images of starch granule with 0% **(A,B)**, 3% **(C,D)**, 6% **(E,F)**, and 9% **(G,H)** HPS additions.

SEM images (Figure 4) showed that the 0% HPS sample consisted of irregular and fractured aggregates, indicating irradiation-induced structural disruption. HPS incorporation smoothed particle surfaces and enhanced interparticle association. The 6% HPS sample exhibited the most compact and homogeneous morphology, with fewer visible defects and voids. By contrast, the 9% HPS sample displayed a rougher surface and localized pores despite its compact appearance, indicating that excessive HPS addition did not further improve structural integrity. These observations suggest that moderate HPS addition was more effective than excessive addition in optimizing the microstructure of the irradiated starch matrix.

### 3.5 Thermal properties

The gelatinization parameters of irradiated rice flour with different HPS additions are presented in Table 4. The gelatinization enthalpy (ΔH) decreased significantly from 4.61 J/g in the control to 3.93 J/g at 9% HPS (*p* < 0.05), indicating a reduction in the thermal stability of crystalline structures. The onset temperature (To) remained relatively stable, while the final temperature (Tc) decreased significantly in the 6 and 9% HPS samples, resulting in a narrower gelatinization temperature range (Tc-To).

**Table 4:** Thermal properties of *γ*-irradiated rice starches with different HPS additions.

Sample	∆*H* (J/g)	Tp (°C)	To (°C)	Tc (°C)
0% HPS	4.61 ± 0.12^a^	58.10 ± 0.17^a^	52.97 ± 1.00^a^	67.83 ± 0.23^a^
3% HPS	4.01 ± 0.06^b^	57.50 ± 0.58^ab^	52.10 ± 0.82^a^	67.03 ± 0.23^a^
6% HPS	3.97 ± 0.12^b^	58.17 ± 0.15^a^	52.23 ± 0.65^a^	65.80 ± 0.66^b^
9% HPS	3.93 ± 0.05^c^	57.03 ± 0.55^b^	53.20 ± 0.95^a^	66.07 ± 0.47^b^

To, onset temperature. Tp, peak temperature. Tc, conclusion temperature. ΔH, gelatinization enthalpy. Values are expressed as mean ± standard deviation (*n* = 3). Values are mean ± SD. Different superscripts within the same column indicate significant differences (*p* < 0.05).

Although XRD results suggest an increase in the proportion of ordered structures, as discussed in Sections 3.3.1 and 3.3.2, the expanded intermolecular spacing at higher HPS levels favors nucleation of a greater number of ordered domains but limits their cooperative packing stability. Consequently, although the overall crystalline proportion increases, individual crystallites are less thermally stable and require less energy to dissociate. This is consistent with the observed reduction in gelatinization enthalpy for hydroxypropylated extruded starch, indicating that less energy is required to disrupt the less ordered crystals (39, 41). The significant decrease in Tc together with the narrowing of the gelatinization range (Tc-To) further supports the presence of a structurally more homogeneous but thermally weaker crystalline population. This narrowing is consistent with the narrower molecular weight distribution observed at higher HPS levels. Recent structural analyses of amylopectin fine structure have demonstrated that molecular weight uniformity is closely linked to the homogeneity of gelatinization thermal transitions (29).

### 3.6 Pasting properties

The pasting properties of irradiated rice flour incorporated with different levels of HPS are presented in Figure 3D and Table 5. The peak viscosity (PV) decreased at 3% HPS, increased significantly at 6% HPS, and decreased again at 9% HPS. This non-linear trend indicates that moderate HPS incorporation promotes viscosity development, whereas excessive HPS weakens the structural integrity of starch granules. The highest PV observed at 6% HPS suggests enhanced granule swelling and paste formation, while excessive HPS (9%) may dilute the native starch matrix and weaken paste structure.

**Table 5:** Pasting properties of *γ*-irradiated rice starches with different HPS additions.

Sample	Peak viscosity (cP)	Breakdown (cP)	Final Visc (cP)	Setback (cP)
0% HPS	296.33 ± 16.50^b^	0.67 ± 1.53^b^	442.00 ± 19.05^a^	146.33 ± 6.66^a^
3% HPS	225.33 ± 6.81^c^	0.33 ± 0.58^b^	335.33 ± 17.64^b^	110.33 ± 12.58^c^
6% HPS	354.67 ± 6.35^a^	4.33 ± 1.53^a^	465.00 ± 18.19^a^	114.67 ± 7.61^c^
9% HPS	221.67 ± 5.51^c^	3.00 ± 1.00^a^	347.33 ± 9.07^b^	128.67 ± 4.16^b^

Values are mean ± SD. Different superscripts within the same column indicate significant differences (*p* < 0.05).

Structural analysis revealed that steric expansion and enhanced hydration promoted granule swelling and amylose leaching, thereby increasing paste viscosity at moderate HPS levels. However, at 9% HPS, the over-expanded molecular conformation and weakened intermolecular cohesion, combined with reduced crystalline thermal stability, made swollen granules more susceptible to shear-induced breakdown (42). Hydroxypropylated starch granules with high degrees of substitution have been shown to exhibit markedly increased shear sensitivity owing to excessive swelling and weakened internal structure (43).

The pasting behavior is also consistent with the chain-length redistribution described in Section 3.2.2. At 6% HPS, the higher proportion of B1 chains reflects a cluster architecture that preserves sufficient intra-granular connectivity to withstand thermal and shear stress during pasting (44). In contrast, the enrichment of short A chains together with the reduction of B1 and B3 chains at 9% HPS indicates weakened cluster connectivity, which reduces granule integrity and limits viscosity development.

Among the tested formulations, the 6% HPS sample exhibited the most favorable pasting characteristics, including relatively high peak and final viscosity together with reduced setback viscosity, suggesting that HPS may inhibit starch retrogradation to some extent. The expanded intermolecular spacing and reduced chain-packing tendency documented throughout the structural analyses (Sections 3.2–3.3) hinder reassociation of amylose and amylopectin chains during cooling, thereby suppressing retrogradation.

Overall, these results demonstrate that HPS regulates the pasting behavior of irradiated rice flour by modulating molecular hydration, amylopectin branch architecture, and intermolecular interactions. Moderate HPS incorporation, particularly at 6%, produces a balanced structural environment that promotes granule swelling while maintaining sufficient granule stability, resulting in the most favorable pasting performance.

### 3.7 Relationship between molecular structure and pasting properties

**Figure 5:**
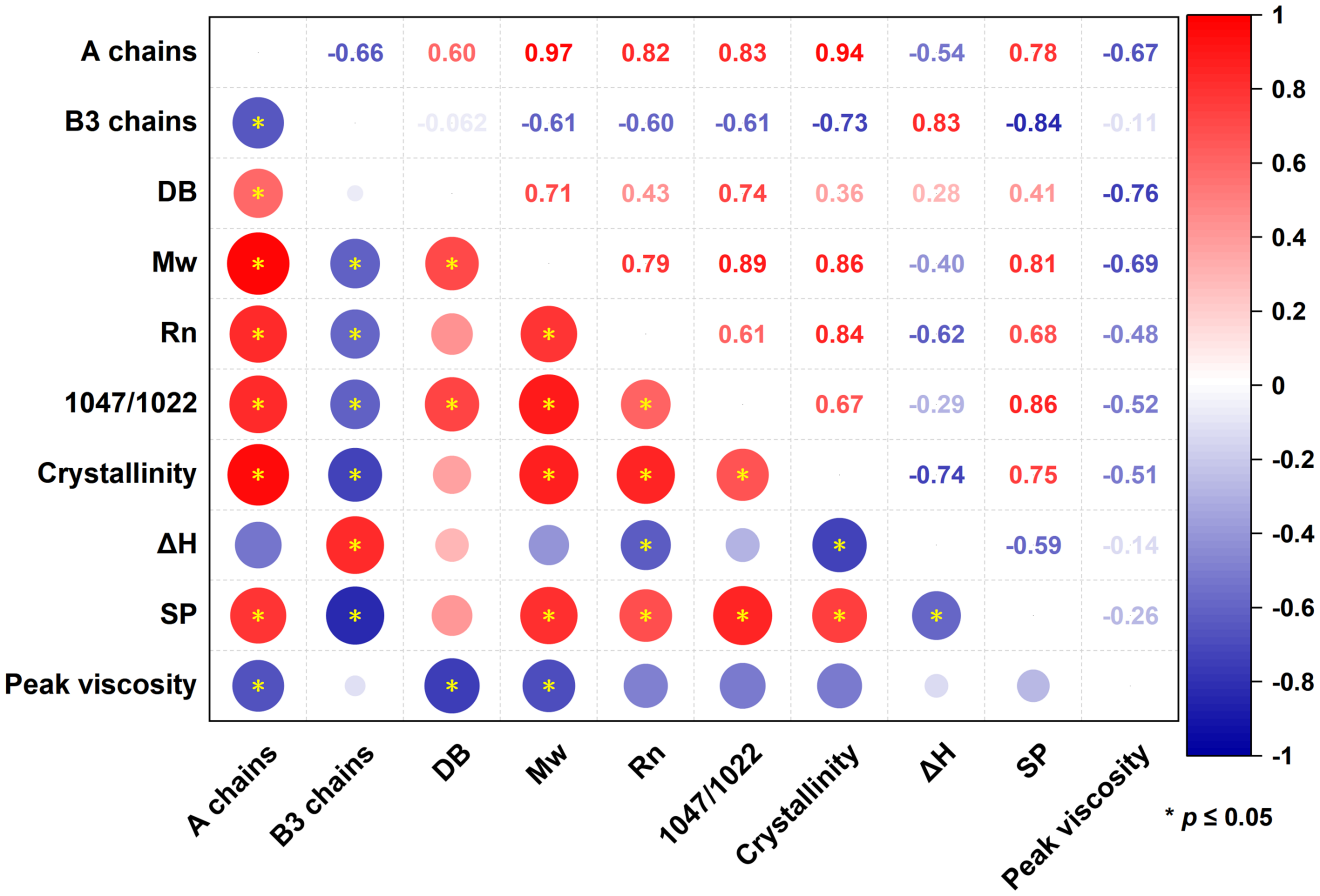
Pearson correlation matrix among structural parameters, thermal properties, and pasting properties of starches.

Pearson correlation analysis was performed to explore the relationships among amylopectin molecular architecture, ordered structures, and functional properties (Figure 5). Significant correlations were observed among structural parameters and physicochemical properties, indicating that HPS-induced structural rearrangements strongly influenced starch functionality.

The proportion of A chains (DP 6–12) showed strong positive correlations with Mw, Rn, 1,047/1022, crystallinity, and SP, indicating that enrichment of short branches enhances molecular flexibility and water uptake, which may promote the formation of short-range ordered structures (Sections 3.3.1 and 3.3.2). However, A chains were negatively correlated with ΔH and peak viscosity, suggesting that excessive short-chain enrichment may weaken crystalline stability and paste network integrity. This trend is consistent with the structural characteristics observed at higher HPS levels (9%), where pronounced short-chain enrichment coincided with reduced paste viscosity.

Conversely, B3 chains exhibited negative correlations with Mw, Rn, crystallinity, and SP, while showing a positive correlation with ΔH. Because B3 chains contribute to inter-cluster connectivity within amylopectin, their reduction may weaken long-range molecular integration and facilitate water penetration into the starch matrix. The relatively moderate variation of B3 chains observed at 6% HPS may therefore help maintain cluster connectivity, which could be associated with the more ordered structural organization and improved pasting properties observed at this level.

Interestingly, ΔH showed a significant negative correlation with crystallinity. As discussed in Section 3.5, this apparent paradox arises because HPS incorporation promotes the nucleation of ordered domains, yet the reduced cooperative packing stability results in crystallites that require less thermal energy to dissociate. Similar trends have also been reported in previous studies, where gelatinization enthalpy decreased with increasing HPS addition, which may suggest the formation of crystalline structures with relatively lower thermal stability (45).

SP was positively correlated with Mw, Rn, 1,047/1022, and crystallinity, whereas peak viscosity showed negative correlations with Mw, consistent with the observation in Section 3.6 that excessive molecular expansion reduces paste network stability during shear. Overall, these correlations indicate that moderate HPS incorporation (6%) is associated with a balanced structural state, in which molecular flexibility and hydration are enhanced while cluster connectivity is largely maintained. In contrast, higher HPS incorporation (9%) may promote stronger short-chain enrichment and molecular expansion, which could disrupt the structural integrity of the starch paste system.

### 3.8 Proposed mechanism for the influence of HPS incorporation on *γ*-irradiated rice starch

**Figure 6:**
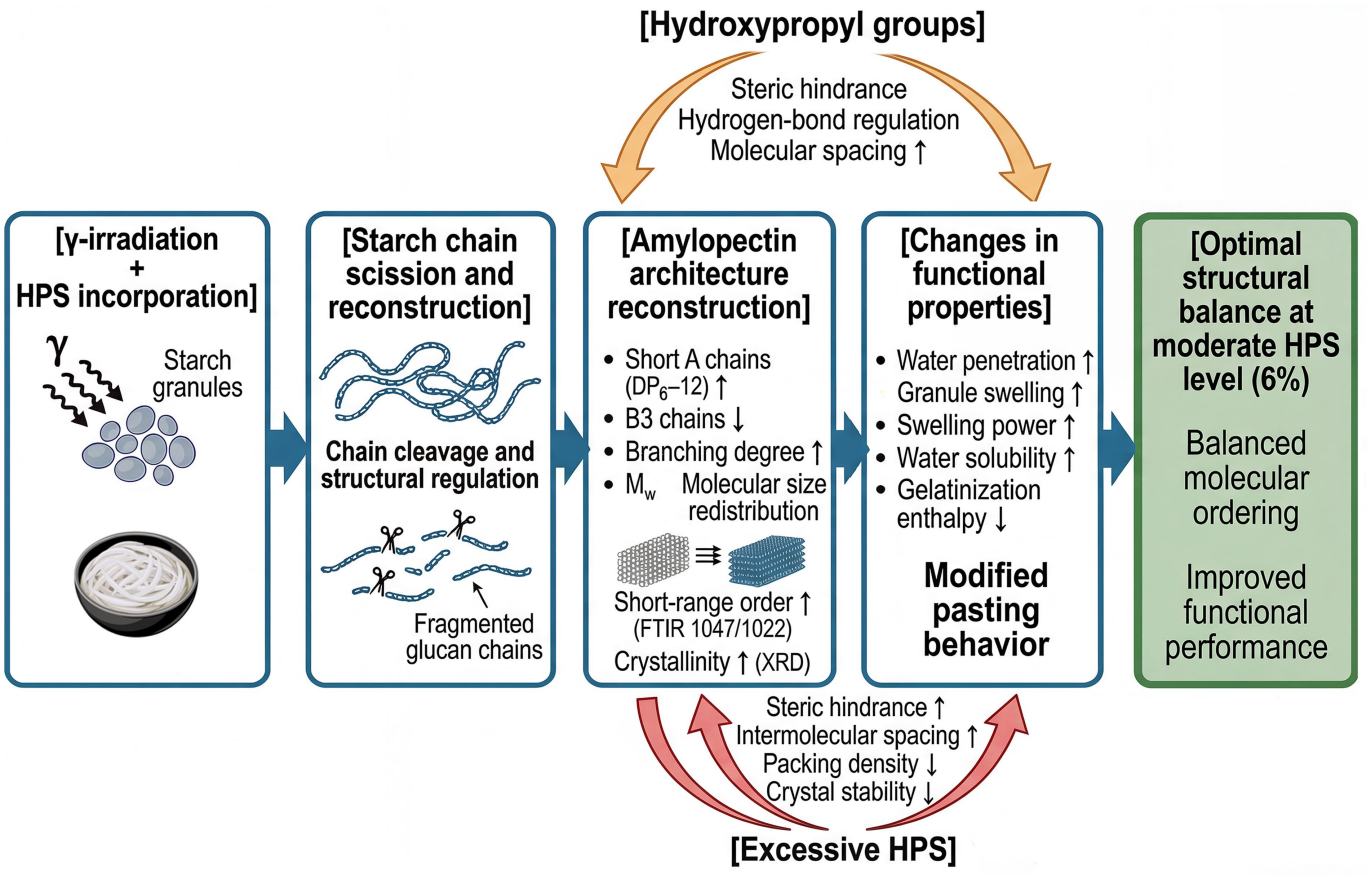
Proposed mechanism for the regulation of structural and functional changes in *γ*-irradiated rice starch by HPS incorporation.

Figure 6 illustrates the proposed mechanism by which HPS incorporation regulates the structure and functionality of *γ*-irradiated rice starch. *γ*-Irradiation induces partial chain scission of starch molecules, generating shorter glucan chains and increasing molecular mobility. Meanwhile, hydroxypropyl substitution increases molecular hydrophilicity and weakens intermolecular hydrogen bonding, thereby facilitating structural rearrangement within the starch matrix (21, 46). These combined effects promote the reconstruction of amylopectin architecture and alter chain length distribution.

Importantly, at moderate incorporation levels (6%), both A chains and B1 chains increased while B2 and B3 chains decreased slightly. This redistribution enhances intermediate branch density without excessive loss of long connecting chains, resulting in a relatively well-integrated amylopectin cluster architecture. Such a structural configuration promotes water penetration and facilitates granule swelling while preserving sufficient granule integrity, leading to improved viscosity development during pasting.

In contrast, excessive HPS incorporation (9%) induced a different structural adjustment in amylopectin. At this level, A chains were markedly enriched, whereas B1 and B3 chains decreased and B2 chains increased. The accumulation of short branches together with the depletion of long connecting chains indicates weakened cluster connectivity and a less integrated amylopectin architecture. Although short chains may enhance hydration, the reduced structural cohesion compromises the integrity of swollen granules, making them more susceptible to thermal and shear disruption during pasting. Meanwhile, the increased proportion of B2 chains may promote molecular reassociation during cooling, contributing to higher setback values.

Overall, the influence of HPS on *γ*-irradiated rice starch can be summarized as a hierarchical regulatory mechanism: the steric and hydration effects reshape molecular conformation of irradiation-fragmented chains, redirect amylopectin branch redistribution, modify short-range ordering and crystalline assembly, and ultimately determine thermal and pasting performance. Moderate HPS incorporation (6%) increases A and B1 chains while slightly reducing B2 and B3 chains, generating a balanced cluster architecture that promotes granule swelling and optimal viscosity development. In contrast, excessive incorporation (9%) enriches short chains and weakens cluster connectivity, resulting in reduced paste stability and increased retrogradation tendency.

### 3.9 Industrial relevance, commercialization considerations, and limitations

From an industrial perspective, HPS can be readily integrated into existing fresh rice noodle formulations, and *γ*-irradiation may serve as a post-packaging non-thermal preservation step. Its practical application will depend on processing throughput, dose uniformity, packaging compatibility, access to irradiation facilities, and regulatory compliance. Compared with refrigeration or freezing, the potential value of *γ*-irradiation is not limited to microbial control. Fresh rice noodles spoil rapidly at ambient temperature, whereas low-temperature storage, although effective in slowing microbial growth, may accelerate starch retrogradation, moisture redistribution, and textural deterioration, thereby reducing eating quality. In this context, *γ*-irradiation may improve microbiological safety while reducing reliance on strict low-temperature storage. Compared with chemical preservatives, this approach is more costly but may be attractive where reduced preservative use is preferred. Therefore, its commercial potential may be greater in large-scale or centralized production systems, particularly where product safety, distribution flexibility, and quality retention are prioritized.

Besides, this study examined the effects of different HPS levels under a fixed *γ*-irradiation condition using a single rice variety. Future studies incorporating a broader control design, including non-irradiated HPS-containing samples, together with additional rice varieties, sensory evaluation, and shelf-life assessment, would provide a more comprehensive understanding of HPS application in irradiated fresh rice noodles. The mechanistic insights presented here are based on integrated structural and physicochemical analyses, and further direct evidence would help strengthen these interpretations.

## 4 Conclusion

This study investigated the effects of hydroxypropyl starch (HPS) incorporation on the structural characteristics and thermal and pasting properties of starch in irradiated rice flour systems. HPS shifted the apparent amylopectin chain-length distribution toward shorter chains and increased the apparent *α* − 1,6 linkage ratio of starch molecules, leading to enhanced short-range molecular order and a slight increase in relative crystallinity, while the gelatinization enthalpy decreased. The addition of 6% HPS produced a more favorable starch structural organization and desirable pasting behavior, characterized by relatively stable molecular conformation, improved viscosity development, and reduced retrogradation tendency. These results indicate that moderate HPS incorporation can effectively regulate the structural organization and functional performance of starch in irradiated rice flour, providing a potential strategy for improving the processing quality of rice flour-based products.

## Data Availability

The raw data supporting the conclusions of this article will be made available by the authors, without undue reservation.
